# The Influence of Mitochondrial Dynamics and Function on Retinal Ganglion Cell Susceptibility in Optic Nerve Disease

**DOI:** 10.3390/cells10071593

**Published:** 2021-06-25

**Authors:** Nicole A. Muench, Sonia Patel, Margaret E. Maes, Ryan J. Donahue, Akihiro Ikeda, Robert W. Nickells

**Affiliations:** 1 Department of Ophthalmology and Visual Sciences, University of Wisconsin-Madison, Madison, WI 53706, USA; nmuench@wisc.edu (N.A.M.); spatel44@wisc.edu (S.P.); Ryan.Donahue@childrens.harvard.edu (R.J.D.); 2 Institute of Science and Technology Austria, 3400 Klosterneuburg, Austria; margaret.maes@ist.ac.at; 3 Boston Children’s Hospital, Harvard Medical School, Harvard University, Boston, MA 02115, USA; 4 Department of Medical Genetics, University of Wisconsin-Madison, Madison, WI 53706, USA; aikeda@wisc.edu; 5 McPherson Eye Research Institute, University of Wisconsin-Madison, Madison, WI 53705, USA

**Keywords:** dominant optic atrophy, glaucoma, metabolism, mitochondria, neurodegeneration, optic nerve, retinal ganglion cells

## Abstract

The important roles of mitochondrial function and dysfunction in the process of neurodegeneration are widely acknowledged. Retinal ganglion cells (RGCs) appear to be a highly vulnerable neuronal cell type in the central nervous system with respect to mitochondrial dysfunction but the actual reasons for this are still incompletely understood. These cells have a unique circumstance where unmyelinated axons must bend nearly 90° to exit the eye and then cross a translaminar pressure gradient before becoming myelinated in the optic nerve. This region, the optic nerve head, contains some of the highest density of mitochondria present in these cells. Glaucoma represents a perfect storm of events occurring at this location, with a combination of changes in the translaminar pressure gradient and reassignment of the metabolic support functions of supporting glia, which appears to apply increased metabolic stress to the RGC axons leading to a failure of axonal transport mechanisms. However, RGCs themselves are also extremely sensitive to genetic mutations, particularly in genes affecting mitochondrial dynamics and mitochondrial clearance. These mutations, which systemically affect the mitochondria in every cell, often lead to an optic neuropathy as the sole pathologic defect in affected patients. This review summarizes knowledge of mitochondrial structure and function, the known energy demands of neurons in general, and places these in the context of normal and pathological characteristics of mitochondria attributed to RGCs.

## 1. Cellular Function, Structure, and Activity of Mitochondria

In order to better understand the role of mitochondria in neurodegeneration, it is important to appreciate the wide variety of functions these organelles have in cells. Since 1949 the role that mitochondria have in generating energy-rich intermediates such as adenosine triphosphate (ATP) has been appreciated [1,2]. The most common representation of mitochondria is as a double membrane bean-like structure. The mitochondrial inner membrane (MIM) is highly convoluted into folds called cristae which are surrounded by the mitochondrial outer membrane (MOM). Energy production is reliant on differential permeability of the MIM and MOM such that energy-rich intermediates generated in the cytosol can be shuttled/transported across the MOM where they interact with proteins embedded in the MIM, allowing for the transfer of high energy electrons (coupled with protons) across the MIM to be coupled with molecular oxygen, resulting in a proton gradient that is then used to generate ATP by oxidative phosphorylation. Mitochondria were thought to arise from aerobic prokaryotes that were ingested by anaerobic proto-eukaryotes (the endosymbiont theory) [3], where they formed a symbiotic relationship with the “host” thereby conferring a selective advantage of being able to neutralize the toxic effects of the growing levels of oxygen in the environment, while also providing greater levels of energy-rich intermediates that could be applied to anabolic reactions. During the course of evolution, mitochondria retained some or all of their own genetic information (mitochondrial DNA—mtDNA), while acquiring components coded from the “host” genome that improved both the efficiencies of detoxification and energy production.

**Figure 1:**
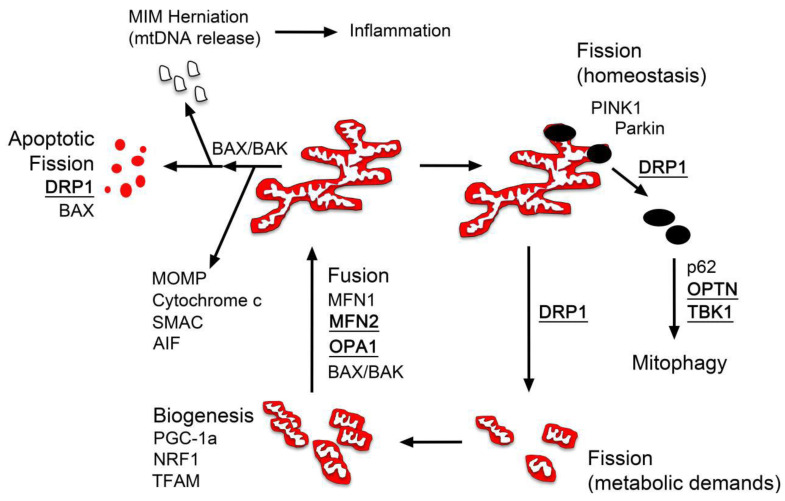
A diagram of mitochondrial dynamics. Mitochondria are in a dynamic flux of fission and fusion, which is determined by factors such as energy/metabolic demands, homeostasis to eliminate damaged or aged organelles, and cell death. This diagram shows some of the relevant gene products that are involved in these processes. Proteins, where mutations within can lead to spontaneous optic atrophy (including Charcot–Marie–Tooth syndrome type 2A) or are associated with familial normal tension glaucoma, are in bold font and underlined (see text). Note that there are three distinct fission mechanisms of reducing mitochondrial size and complexity (homeostasis, metabolic demands, and apoptosis). Abbreviations: MIM, mitochondrial inner membrane; MOMP, mitochondrial outer membrane permeabilization; mtDNA, mitochondrial DNA.

To appreciate mitochondria as small “bean-shaped” organelles is misleading, however. In realty, mitochondria are diversely shaped organelles that exist as a network that is constantly in a process of growth and reduction in size, which is a reflection of both the environment and energy demands that are placed on a cell and the need to recycle aged or damaged regions of these organelles. Collectively, the constant remodeling of mitochondria is termed mitochondrial dynamics. Individual mitochondria can vary dramatically in size depending on the cell type [4] and larger mitochondria are often highly reticulated, which may enhance energy distribution to regions of high demand through membrane potential conduction along the reticulum [5]. Figure 1 shows a diagram of several of the processes that are involved in mitochondrial dynamics. A variety of genes have been identified as being key in both the increase (fusion) and the decrease (fission) in mitochondrial size. Several of the genes that are relevant to this discussion of mitochondrial involvement in neurodegeneration, particularly with respect to the stability of the optic nerve, are listed in the figure. Excellent review articles on mitochondrial dynamics are numerous and for the purposes of this discussion we will provide only an overview of the processes of fusion, fission, and mitophagy. We recommend the following reviews for further reading [6,7].

Mitochondrial fusion leads to larger and more reticulated mitochondria. Fusion occurs in response to changes in demand for oxidative phosphorylation and is also thought to increase resiliency of mitochondria by the combining of organelles with weaker function (i.e., reduced membrane potential caused by aging) with more robust ones [6]. This process has been reported in cells undergoing mild stress and may help to reduce the need for an increase in mitochondrial clearance. Orchestration of fusion is mediated by dynamin-like GTPases called mitofusins (MFN1 and MFN2), which mediate MOM combination, and optic atrophy 1 (OPA1), which facilitates MIM fusion. OPA1 also helps form and maintain cristae structure and has also been reported as a tether for mtDNA to facilitate its distribution [8]. Mutations in OPA1 cause atrophy of the optic nerve and increased risk of developing normal tension glaucoma [9], which will be discussed in more detail below. No disease phenotypes have been reported for mutations in MFN1 (reviewed by [7]), suggesting that it may be critical for development resulting in embryonic lethality, but mutations in MFN2 are the principal cause of Charcot–Marie–Tooth syndrome type 2A [10], which is also associated with optic neuropathy [11]. Additionally, mitochondrial fusion is influenced by interactions with members of the BCL2 gene family. Anti-apoptotic BCLX_L_ has been reported to enhance fusion and increase mitochondrial biomass in neurons [12], while studies using pro-apoptotic BAX deficient cells indicate that this protein is essential for mitochondrial fusion in normal conditions [13,14]. Interestingly, mutations in BAX which destroy its normal function in cell death can rescue mitochondrial fusion in deficient cells [4], suggesting that its role in fusion is by a mechanism that is distinct from its function during apoptosis.

Mitochondrial fission is the process of fragmenting regions of mitochondria that have reduced function, principally induced by stress and age, but can also be induced by changing energy demands within the cell and during cell division (reviewed by [6]). The main protein required for fission is also a dynamin-like GTPase called DRP1 (*DNM1L*). DRP1 is normally cytosolic, but is recruited to fission sites by other adaptor proteins (MiD49, MiD51 and MFF in mammalian cells), where it is thought to create a constrictive ring around the mitochondrion [15,16]. DRP1-deficiency results in the formation of highly elongated tubular mitochondria, indicative of an imbalance between fusion and fission. Several studies have suggested that blocking DRP1 function is protective for cells during periods of stress [17,18,19,20,21,22]. Nevertheless, mutations in DRP1 have also been reported as causative for dominant optic atrophy [23], underlying the sensitivity of the optic nerve to abnormalities in mitochondrial dynamics.

Damaged or aged mitochondria are targeted for degradation through macroautophagy or mitophagy. Elucidation of this pathway was driven by the identification of genetic loci associated with familial Parkinson’s Disease leading to the discovery of both PTEN-induced putative kinase (PINK1) and Parkin. In healthy mitochondria, PINK1 is normally internalized across the MOM, where it is initially cleaved by the mitochondrial processing peptidase (MPP) [24] and then by presenilin-associated rhomboid-like protein (PARL). Organelles with increased levels of reactive oxygen species (ROS), unfolded proteins, and/or reduced membrane potential, however, fail to import PINK1, which begins to accumulate on the MOM surface. In this location, PINK1 phosphorylates Ser65 of ubiquitin, facilitating the recruitment of the E3 ubiquitin ligase Parkin. PINK1 also phosphorylates Ser65 of the ubiquitin-like domain of Parkin, activating its ligase activity resulting in further ubiquitination of a variety of substrates on the MOM. These ubiquitinated sites are also phosphorylated by PINK1, leading to the rapid coating of the mitochondria in phosphorylated ubiquitin chains [25]. Once highly ubiquitinated, the mitochondria are targeted for assembly into autophagosomes through the interaction with several linker proteins, including p62 and optineurin (OPTN) [26]. Of relevance to a discussion of the role of mitochondria in optic neuropathies, the activity of OPTN as a linker protein is enhanced by being phosphorylated by TANK-Binding Kinase 1 (TBK1), which increases both ubiquitin-OPTN binding affinity [27] and OPTN-LC3 binding affinity [28]. Both OPTN and TBK1 have been linked to familial normal tension glaucoma [29,30,31,32,33]. While mitophagy is considered a cell-intrinsic mechanism for mitochondrial clearance, mitochondria can also be cleared by a cooperative mechanism between cells in a process termed transmitophagy [34]. In this case, aged or damaged mitochondria, associated with high levels of PINK1, Parkin, and ubiquitin, are packaged into vesicular evulsions that are taken up by adjacent cells for degradation [35]. This process may play an important role in the normal physiology, and disease, of mitochondria in neurons with long projection axons and will be discussed in detail below. Lastly, it is important to distinguish the process of normal mitochondrial dynamics and clearance, from mitochondrial fragmentation during apoptotic cell death. In the latter, mitochondria become rapidly fragmented in a process that requires the activity of both DRP1 and BAX, and interaction with constricting processes from the endoplasmic reticulum [4,36,37,38,39,40]. These mitochondrial remnants are packaged with other degraded organelles and released as apoptotic bodies which are cleared by either professional or surrogate macrophages [41,42].

Energy demands are also thought to drive mitochondrial distribution within the same cell, such that regions with higher energy demand contain the highest concentration of mitochondria. The cellular distribution of mitochondria is regulated both by transport and tethering mechanisms [43]. Transport is regulated by the molecular motors kinesin (KIF5 isoforms) and dynein, which are linked to mitochondria through protein complexes principally involving Mitochondrial Rho GTPase (MIRO1) (reviewed by [44]). How mitochondria are targeted to areas of high energy demand is a subject of intense study. Reports suggest that regions of high ADP:ATP ratios reduce kinesin activity possibly by the competitive binding of ADP to the motor. Regulation of MIRO1 also plays an important role in targeting mitochondria, which is mediated by regions of elevated Ca^2+^ ions. Ca^2+^ interacts with MIRO1 and causes its dissociation from kinesin [45]. Once immobilized, mitochondria become anchored to cytoskeletal elements by syntaphilin (SNPH) in a process that may be regulated by SNPH ubiquitination. Rather than inducing SNPH degradation, ubiquitination stabilizes SNPH binding to microtubules [46]. To date, the majority of investigation on mitochondrial targeting and the role of SNPH has been conducted in neurons and cancer cells, and it is unclear if SNPH is a ubiquitous anchoring protein in other cells.

Mitochondrial stabilization may also be facilitated by membrane contact sites (MCSs), which are specialized microdomains where the membranes of two organelles are in close contact with each other (reviewed in [47,48,49]). To be defined as an MCS requires that the two organelle membranes are distinct and not fusing (thereby having unique proteomes and lipidomes between the organelles) and are kept in contact by one or more tethers. Additionally, the MCS must have distinct biological functions. Mitochondria and the endoplasmic reticulum form MCSs that have a variety of functions important for cell homeostasis and death, including lipid transfer and facilitating apoptotic mitochondrial fragmentation. A third important role is transfer of Ca^2+^ from the endoplasmic reticulum to the mitochondria, which is essential for several mitochondrial activities including oxidative phosphorylation. Transfer is mediated by inositol 1,4,5-triphosphate receptors (IP3Rs) in the ER membrane, the voltage-dependent anion channel 1 (VDAC1) in the MOM, and the chaperone glucose regulated protein 75 (GRP75). These proteins are also considered as a principal tethering complex for MCSs involving these two organelles. Under normal homeostatic conditions, excess Ca^2+^ in the mitochondria is released and taken back up into the endoplasmic reticulum through activity of the sarcoplasmic reticulum/ER ATPase (SERCA) Ca^2+^ channel, but stress can dramatically change Ca^2+^ regulation at these sites resulting in cellular pathology (see below). In addition to the Ca^2+^ channeling proteins that comprise the MCS, Ca^2+^ regulation can be modified by anti-apoptotic proteins of the BCL2 gene family such as BCL2 and BCLX_L_. These proteins can reportedly bind directly to both the IP3Rs and VDAC1 through the 4th BCL2 Homology (BH4) domain to modulate their activity [50,51,52].

## 2. Mitochondria in Cellular Pathology

The various contributions of mitochondria in cellular pathology can be grossly classified into categories of passive effects and active effects. Passive contributions include features of these organelles that lead to impairment of respiratory activity, such that cells are unable to meet challenges they face during times of stress. This effect is primarily attributed to defects in mitochondrial dynamics and encompasses genetic mutations in genes involved in fission and fusion, as well as mitophagy. Many of these defects manifest as inherited diseases, many of which often directly, and sometimes exclusively, lead to optic neuropathies. Passive defects can also include alterations or deficiencies in mitochondrial transport and tethering, such that these organelles are not appropriately localized to cellular compartments that have high ATP demands [53]. Transport deficiencies have been implicated in a variety of neurological disorders (reviewed by [53,54]). Lastly, there is a substantial body of literature showing that aging is associated with reduced respiratory capacity and accumulation of damaged mitochondria as a result of reduced mitophagy [55,56].

Mitochondria actively contribute to cellular pathology through their role in mediating the intrinsic apoptotic program by interacting with proteins of the BCL2 gene family. As noted above, while both pro- and anti-apoptotic members of this family are active in regulating normal mitochondrial dynamics, during occurrences of terminal stress, pro-apoptotic members BAX and BAK are stimulated to aggregate at the MOM where they undergo a conformational change, allowing them to insert into the membrane and begin to form homodimers. Studies of this process have shown that molecules such as BAX can initiate membrane destabilization, leading to proteolipid pores [57] large enough for pro-apoptotic signaling molecules (such as cytochrome c) to exit the intermembrane space between the MOM and MIM and enter the cytosol [58]. A major site of BAX and BAK aggregation occurs at the MCSs between the endoplasmic reticulum and the MOM. During periods of stress, an increase in the unfolded protein response (UPR) of the endoplasmic reticulum leads to the accumulation of more MCSs with mitochondria and an increase in Ca^2+^ transfer. Part of this is thought to drive increased ATP production (by stimulating pyruvate entry into the TCA cycle and oxidative phosphorylation) to fuel the UPR, but it can also lead to an overload of Ca^2+^ and eventually induction of apoptosis. This latter process is associated with disassembly of OPA1 complexes and DRP1-mediated transfer of cardiolipins from the MIM to the MOM which, in turn, facilitates the release of cytochrome c during the formation of pro-apoptotic BAX-mediated pores [59]. Cytochrome c itself is a critical activator of the apoptosome, which is a structure containing procaspase 9 [60,61]. Once caspase 9 is autolytically cleaved to an active form, it is able to recruit and activate procaspase 3, leading to activation of the caspase proteolytic cascade [62]. Continued aggregation of BAX into oligomers leads to larger pore structures in the MOM [63,64,65] allowing larger molecules to leak out [66,67,68]. These macropores become so large, in fact, that regions of the MIM become extruded through the pores, ultimately leading to extra-mitochondrial presentation of mtDNA [69] that contributes to mitochondrial damage associated molecular patterns (DAMPs) that further exacerbate pathological effects including recruitment of the innate immune response [70,71]. BAX oligomers are also implicated in the process of apoptosis-induced mitochondrial fragmentation, where they may participate with other fission proteins, such as DRP1, in this process [4].

The consequences of mitochondrial dysfunction during intrinsic apoptosis reach further than just the activation of the caspase cascade. Disruption of the MOM and loss of cytochrome c likely result in a significant inhibitory effect on oxidative phosphorylation leading to reduced ATP production and the generation of ROS by incomplete transfer of high energy electrons to molecular oxygen, which would further damage the cell. Several studies have shown, for example, that blocking elements of the caspase cascade using inhibitors, provides only a transient protective effect in cells undergoing intrinsic apoptosis [72,73]. The actual link between mitochondrial dysfunction associated with pro-apoptotic BAX and BAK activation and the production of ROS is not absolute, however. Several studies have reported an increase in ROS (superoxide) release in response to activation of pro-apoptotic BCL2 family proteins [74,75], including a clear association with the generation of cytosolic superoxide after BAX recruitment to the MOM [76]. Alternatively, mitochondria are not the only potential source of ROS in a cell [77] and it is not possible to generalize that all ROS-based pathology is linked to mitochondrial dysfunction.

## 3. Mitochondria in Normal Neuronal Activity and Neurodegeneration

The importance of mitochondria in neuronal health and disease is an area of intense investigation. Mitochondrial dysfunction, alterations in mitochondrial dynamics, and reduced mitochondrial transport have all been implicated at some level in a wide variety of neurological disorders [78] including (but not limited to) Alzheimer’s disease [79], Parkinson’s disease [80,81], multiple sclerosis [82], amyotrophic lateral sclerosis [83], Huntington’s disease [84], seizures [85], dominant optic atrophy [86], and peripheral neuropathies such as Charcot–Marie–Tooth disease [10]. The contributory role of mitochondria to neurodegeneration is not surprising given that 20–25% of the daily energy consumed in humans is by the brain, which comprises 2% of the body mass [87].

Neurons execute a plethora of ATP-requiring activities, but the predominant consumers are recovery of action potentials and synaptic activity [88,89,90]. Action potential energy demands center around the rebalancing of intracellular Na^+^ and extracellular K^+^ ions after firing, which is mediated by the activity of the Na^+^/K^+^ ATPase pump. Calculations for ATP to complete an action potential are variable in the literature and are different depending on the presence or absence of myelin. It is estimated that the energy expenditure is from 10^11^ to 10^12^ ATP molecules/cm^2^/action potential of cell membrane in non-myelinated axons, with a minimum of 10^6^ molecules required to rebalance ion concentrations at nodes of Ranvier [91]. Empirically, the data show that 3.3 × 10^8^ molecules of ATP are required for balancing an action potential for a single cortical pyramidal neuron with a myelinated axon and 5.4 × 10^8^ molecules of ATP for a hippocampal neuron with an unmyelinated axon, when the axon lengths are set to 4 cm [92]. Attwell and Laughlin [88] similarly estimated that an average neuron expends 3.84 × 10^8^ molecules of ATP per action potential.

While recovery of action potentials is energy consuming, estimates suggest that synaptic function may require even greater consumption of ATP [90,93]. Breaking down pre- and post-synaptic energy requirements for a single vesicle event in a glutaminergic synapse yields an energy cost in the range of 1.64 × 10^5^ ATP molecules/vesicle, and although a portion of this cost is transferred to astrocyte function to recycle glutamine, this accounts for only ~3% of the energy expenditure [88,89]. Metabolically intact synapses have a reservoir of ~10^6^ molecules of ATP [93] and local ATP production is driven by synaptic activity. There is some controversy on how this ATP is supplied, particularly in times of high activity. Estimates suggest that each synapse has an average of a single mitochondrion localized to it [94] and that the majority of ATP production (~93%) is generated by oxidative phosphorylation. Conversely, some data indicate that higher rates of activity are not associated with an increase in O_2_ uptake, suggesting that localized glycolysis is activated to provide ATP for the increase in energy demand (reviewed by [89]).

Intracellular transport is also a high energy consuming process in neurons. Transport mechanisms have best been studied in axons and are comprised of both fast (~1 µm/s) and slow (~0.1 µm/s) speeds. Fast transport is generally attributed to movement of organelles and vesicles, including mitochondria. Small molecules such as proteins and mRNAs move by slow transport. In neurons, approximately 20–30% of the mitochondria are actively being transported. In axons, which contain directional microtubules (with the growing end localized to the axon terminus), mitochondria are moved in the retrograde direction by dynein, and in the anterograde direction by kinesins (reviewed by [90]). Transport in the processes of the dendritic arbor is less well-defined, but mitochondrial motility may be principally controlled by dynein motors, which make use of mixed directional microtubules (reviewed by [54]). ATP consumption for axonal transport has been calculated based on the stoichiometry of motor proteins for a single vesicle (i.e., 1–2 kinesin proteins and 6–12 dyneins/vesicle), motor step size, and rate of ATP hydrolysis. These calculations suggest that transport of a single vesicle along the length of a 1 m human motoneuron axon would consume 1.25 × 10^8^ molecules of ATP for anterograde transport and 7.5 × 10^8^ molecules for retrograde transport [54]. Importantly, transport mechanisms of mitochondria are highly coordinated in neurons, so that these organelles become stabilized in regions of high metabolic activity. This coordination is modulated by several factors including cytoskeletal elements localized to axonal hillocks and mitochondrial dynamics. In the latter, studies show that inhibiting the fission protein DRP1 leads to a loss of motility of these organelles in the neuronal soma. As indicated above, the localization of mitochondria in neurons is regulated by microenvironments including ADP:ATP ratios, increased levels of intracellular Ca^2+^, and elevated glucose, all features of active synapses [54,90], and the anchoring function of SNPH.

Stress, at least in early stages, can increase the rate of mitochondrial transport in neurons [95]. In some cases, mitochondria are transported to regions of damage such as axotomized axons and there is a correlation between the mobilization of mitochondria and the ability of the axon to regenerate [96,97,98]. Alternatively, distal mitochondria are frequently significantly aged [99] and present a liability to the stressed cell. There is evidence that distal (axonal) mitochondria are not cleared by localized mitophagy. Some neurons exhibit a dramatic shift in retrograde transport of these organelles during conditions of mild stress. In this case, there is a bulk release of mitochondria from SNPH [100] followed by their transport to the soma, where they are targeted for removal by mitophagy [101].

## 4. Fuel Sources for Neurons

While the majority of ATP production in neurons is sourced from mitochondria, neurons can also supply energy directly from aerobic glycolysis using glucose as a substrate. However, the vast majority of energy metabolites are provided to neurons in the form of pyruvate and lactate, which are generated by adjacent glial cells. Several studies, including those using mouse optic nerves ex vivo, suggest that astrocytes are the principal source of these metabolites. These cells break down stores of glycogen and pass lactate through the so-called Astrocyte to Neuron L-lactate shuttle (ANLS) using the monocarboxylate transporter MCT1 [102,103,104]. The contribution of the ANLS in white matter is a subject of controversy, however, given the limited access of astrocytes to axons wrapped in the oligodendrocyte myelin sheath [90]. Instead, oligodendrocytes appear to be the main source of lactate for axons [90,105,106] and these cells are also highly enriched for the MCT1 transporter. Unlike astrocytes, however, which catabolize glycogen to generate metabolic intermediates [102,103,104,107], pyruvate and lactate support from oligodendrocytes in the optic nerve is activity driven, with the release of glutamate stimulating glucose uptake, glycolysis, and lactate export in oligodendrocytes [108]. Similarly, astrocytes may not be the primary, or only, energy supporter for neurons in the inner retina, including retinal ganglion cells. Müller cell glia have been shown to be actively involved in lactate homeostasis, including selectively using lactate as an energy source, while exporting lactate in times of retinal stress [109].

## 5. Mitochondrial Localization within Retinal Ganglion Cell Architecture

**Figure 2:**
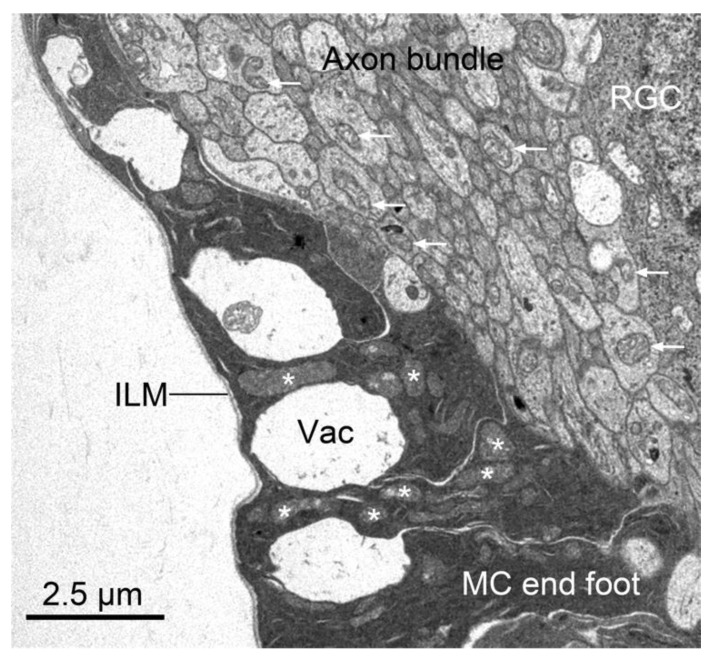
Müller cell end feet surround axon bundles in the nerve fiber layer. Transmission electron micrograph of a region of the nerve fiber layer of the mouse retina. In this image, the axons are sectioned transversely, appearing round. They are easily identified by the multiple microtubules arranged throughout the axoplasm. Examples of mitochondria within axons are highlighted with arrows. Müller cells (MC) extend electron-dense processes (end feet) all the way to the inner limiting membrane (ILM) and there are often large vacuole-like structures (Vac) that probably form between separate end feet, although this may be a fixation artifact. Examples of mitochondria, which are densely packed into each end foot, are highlighted with asterisks. An adjacent retinal ganglion cell (RGC) is present in the top right corner of the image.

While mitochondrial dysfunction is widely appreciated in the process of neurodegeneration, retinal ganglion cells (RGCs) may be the most vulnerable of all neuronal cell types. RGCs are long projection neurons of the CNS, with axons that can track 50–100 mm to visual centers in the brain in primates [110,111]. RGC somas reside in the innermost layer of the retina facing the vitreous humor. They receive neuronal input from both amacrine and bipolar neurons [112] by extending complex branched dendritic arbors which stratify in different levels of the inner plexiform layer (the OFF and ON sublaminae). Axons of the RGCs project across the surface of the inner retina to form the nerve fiber layer. They extend in bundles to the optic nerve head (ONH) where they make an abrupt ~90° turn and pass through the scleral canal and into the optic nerve. Additionally, RGCs are contacted by astrocytes residing along and within the nerve fiber layer and Müller cell glia both in the inner plexiform layer and in the nerve fiber layer, where Müller cell end feet wrap around bundles of axons (Figure 2). In most mammals, intraretinal axons are unmyelinated, a feature thought to provide greater transparency to the retina, since the light path has to penetrate through it to reach the photoreceptor layer.

The ONH is specially engineered to provide structural support to the pressurized globe and is associated with columns of glial astrocytes in mice (the glial lamina) [113,114,115], vertically oriented collagen and elastin beams associated with astrocytes in the rat [116] or horizontally oriented collagen and elastin beams wrapped in astrocytes that form pores in larger vertebrates (the lamina cribrosa) [117,118,119,120], to allow exit of the bundled axons as they enter the optic nerve. A feature unique to the central nervous system is found in the lamina cribrosa where the astrocytes reside outside the laminar beams and do not make direct contact with blood vessels that course inside the beams. This implies a significant barrier to the astrocytes to access nutrients and O_2_ in this region. The lamina cribrosa in large vertebrates, however, also contain a unique cell type called the lamina cribrosa cell [118,120] and recent morphometric studies have indicated that these cells extend long processes into the beams to make contact with the central blood vessel [120]. Distal to the laminar region, axons enter a zone where they begin to become myelinated (the myelin transition zone—MTZ) and then reach a point where they become fully myelinated.

The ONH plays a central role in the pathology of optic neuropathies, such as glaucoma, and represents a site of extreme strain under conditions of intraocular pressures that exceed physiological parameters [121,122,123]. Glial cells in this region undergo dramatic molecular and morphological changes in response, which are characterized by activation of the astrocytes and remodeling of the connective tissue components [124,125,126,127,128,129]. Importantly, this site is considered the initial site of damage in glaucoma [115,130] and is characterized by a region of blockage of both anterograde and retrograde axonal transport [131,132,133,134,135]. There are also several important changes in mitochondrial biology associated with pathology to the ONH in glaucoma, which will be discussed in more detail below.

**Figure 3:**
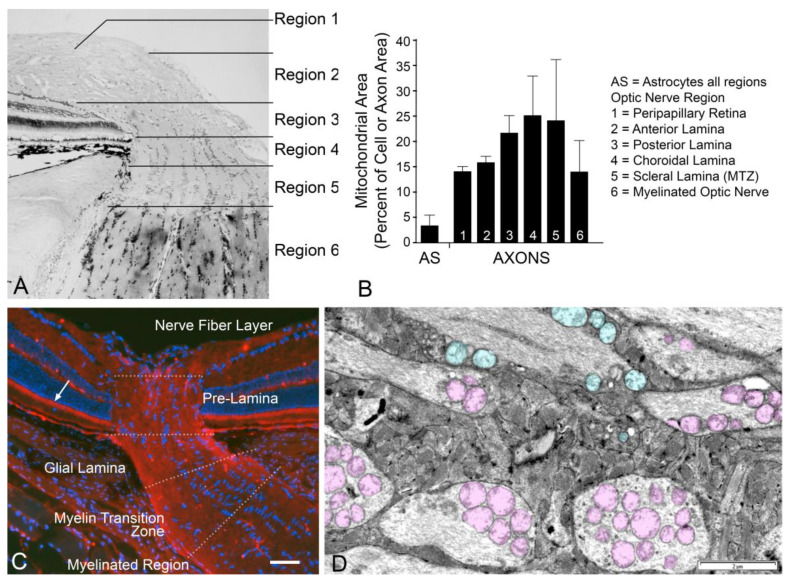
RGC axons in the optic nerve head contain a high concentration of mitochondria. (**A**) A histologic section through the optic nerve head of a non-human primate. Regions that were identified for a detailed ultrastructural analysis are indicated. (**B**) Graphic representation of data presented in [136] of mitochondrial area as a percentage of axoplasmic area, or cell area for astrocytes (AS), in each region of the optic nerve head. These data were originally presented in table form and are reproduced with permission from Elsevier Press. (**C**) Optic nerve section of a mouse optic nerve head, immunostained for the mitochondrial protein TOM20 (see supplemental methods). Different regions of the nerve head and adjacent regions are indicated. Note the high concentration of mitochondrial staining in the pre-laminar region, the glial lamina and the myelin transition zone (MTZ in B), as well as the photoreceptor cell inner segments (arrow). Scale bar = 100 µm (approx.). (**D**) Transmission electron micrograph of a cat optic nerve head in a region showing close apposition of unmyelinated axons and astrocytes. Mitochondria within axons are pseudo-colored pink while mitochondria in adjacent astrocytes are pseudo-colored teal. Image courtesy of Drs. Kazuya Oikawa and Gillian McLellan. Scale bar = 2 µm.

The majority of investigation has focused on mitochondria in RGC axons at the ONH. Pioneering studies on the ONH of the Rhesus monkey revealed that the density of mitochondria in RGC axons (measured as a function of axon area in electron micrographs) is greatest in the regions of the lamina and MTZ [136] (Figure 3), and reaches levels five times greater than mitochondrial density within glial cells in this region. Similarly, mitochondrial density is high in this region of the mouse glial lamina. The reasons for such a high density of mitochondria in this region are not entirely clear, but the implication is that this is an area of high energy demand for RGCs. Since there is no synaptic activity in this region, an explanation that has been commonly posited is that RGCs require the energy to balance the higher cost of action potentials in these segments of unmyelinated axons. This reasoning, however, does not account for the nearly two-fold difference in mitochondrial density found in RGC axons within the laminar/MTZ regions compared to unmyelinated axons in the nerve fiber layer. It is possible that energy requirements are supplemented, or energy demands are modulated, by the high concentration of both astroglia and Müller cell end feet that envelop the axons in the nerve fiber layer. Alternatively, studies suggest that axons with 90° bends require higher excitation thresholds [137], although it is not clear if this impacts energy requirements distal to the bend.

An early hypothesis suggested that the greater concentration of mitochondria in the ONH represented a region of impaired transport [136] which created a bottleneck for materials being transported in the axon. This theory has largely been abandoned, in part by axonal transport studies showing no signs of material accumulation in normotensive eyes [131,138]. This region, however, also marks the point at which axons traverse a pressure gradient defined by the IOP on one side and the combined pressures of the cerebrospinal fluid (CSF) and vascular compartments on the other (creating the “translaminar pressure gradient”). Several studies suggest that pressure gradients negatively affect axonal transport [139], and are sites of increased expression of mitochondrial genes [140], indicating increased energy requirements of axons that cross them to function normally. Changes in the translaminar pressure gradient are associated with increased risk of developing glaucoma, particularly in patients with otherwise normal IOP ranges (normal tension glaucoma), who reportedly have CSF pressures below normal ranges [141,142]. Experimentally, an optic neuropathy can be induced in non-human primates with artificially lowered CSF pressure [143]. Taken together, these observations suggest that the high concentration of mitochondria in the ONH is a consequence of an energy requirement needed for axonal transport rather than the conventional high energy users in neurons of synaptic function and balancing of ions after an action potential.

The MTZ in the ONH of mice has also been identified as a region of active transmitophagy. Astrocytes, but not microglia, in this region selectively express the phagocytosis-related gene Mac-2 and ultrastructural analysis indicates that they are actively engulfing large evulsions from adjacent RGC axons [144]. Serial blockface scanning electron microscopy and studies using a tandem-fluorescent reporter targeted to mitochondria in RGCs, showed that the evulsions contained clusters of mitochondria. Once internalized, these evulsions entered the astrocyte lysosomal pathway [145]. This process appears to be the primary mechanism of mitochondrial clearance in RGCs, exceeding the level of cell-intrinsic mitophagy in the RGC soma. Importantly, transmitophagy was evident even in healthy eyes, but during ocular hypertension-induced stress, the level of Mac-2 expression increased commensurate with an increase in the localization of the axonal damage marker phosphorylated neurofilament [144]. During IOP-induced stress, there was also an increase in the amount of insoluble (protease resistant) γ-synuclein (SNCG) present in the evulsions. SNCG may be an important regulator of the transmitophagy process, since IOP-induced elevations in astrocytic Mac-2 expression were impaired in *Sncg*^−/−^ mice [144]. Interestingly, *Sncg*^−/−^ mice also exhibited greater levels of damaged axons in these experiments, suggesting that active transmitophagy may be an important damage response mechanism for RGCs.

Mitochondria in other compartments (i.e., soma, dendritic arbors, and pre- and post-synapses) of RGCs have been less well-studied. Dendritic arbor structure and remodeling is a particularly exciting field of research in RGC pathology. Both non-human primate and mouse models of glaucoma and acute optic nerve injury indicate that RGC dendritic arbors exhibit dramatic decreases in arbor complexity and shrinkage of their dendritic field [146,147,148,149,150,151], particularly in the OFF sublamina of the inner plexiform layer [152] in a process that is associated with the loss of both pre- and post-synaptic connections [153]. The susceptibility of OFF RGCs also extends to ON-OFF RGCs, which exhibit arbors that separately ramify in the ON and OFF sublaminae of the inner plexiform layer, respectively. In a mouse model of ocular hypertension these cells exhibit greater degenerative changes selectively in the OFF arbor regions [152]. Dendritic arbor complexity and growth is fundamentally influenced by both mitochondrial activity and placement. Studies using rat hippocampal slices or primary cultures showed that mitochondrial density was highest in developing dendritic spines of CA1 pyramidal neurons, but then dropped off after completion of arbor development [154]. Altering mitochondrial dynamics by either impairing or increasing the activity of DRP1 had the effect of reducing or increasing, respectively, the accumulation of mitochondria in arbor projections [154]. Similarly, germ-line deletion of Miro1, the linker protein between kinesin motors and mitochondria, negatively affected both the localization of mitochondria to hippocampal neuronal arbors, and their normal development [155]. In developing *Drosophila* larvae, over-expression or loss of function of the mitochondrial Prel protein, which is normally involved in the transfer of phosphatidic acid between the MIM and the MOM, leads to mitochondrial fragmentation. Concomitantly, modulation of Prel resulted in the abnormal development of arbors in class IV “dendritic arbor” neurons [156]. Mitochondrial depletion in the dendritic arbor may also play a role in pathological remodeling. Elegant experiments in which Miro1 was selectively deleted in mature neurons resulted in arbor shrinkage and eventual neurodegeneration [155].

**Figure 4:**
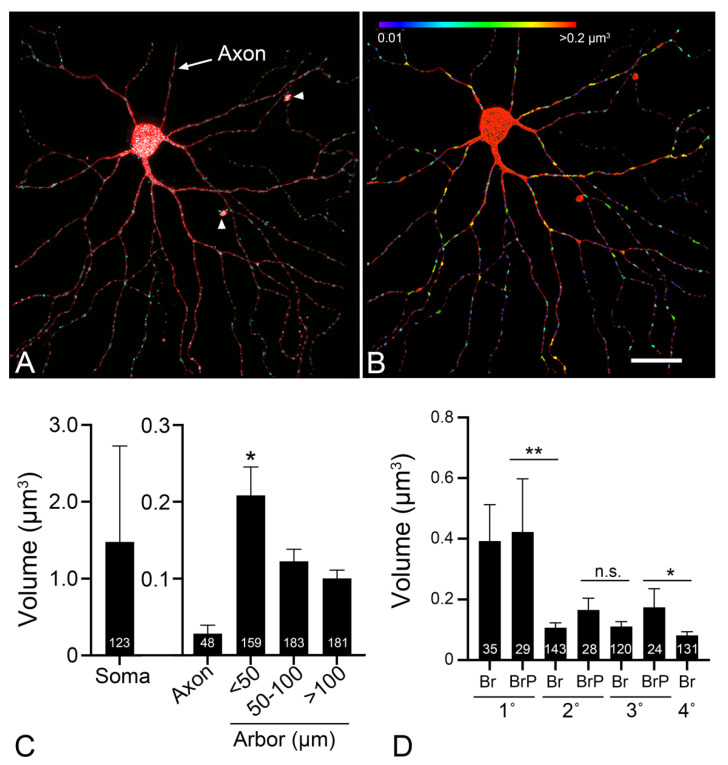
Mitochondrial distribution in a mouse ON-αRGC. Masked confocal Z-stack image of a single RGC from a transgenic mouse expressing Thy1-mitoCFP to identify mitochondria. The cell was labeled by biolistic transfection with a plasmid expressing tdTomato. (**A**) Merged image showing the cell fill and CFP-positive mitochondria. The intraretinal axon of the cell is identified. Additionally, the dendritic arbor exhibits two evulsions (arrowheads) that contain mitochondria (see Figure 6). (**B**) Mitochondrial volume was analyzed using the surface function of Imaris and a heat map of mitochondrial sizes was rendered. Size bar = 25 µm. (**C**) Bar graph (mean ± standard deviation) of mitochondrial volume as a function of distance from the center of the soma in the RGC shown in (**A**). The numbers of mitochondria identified in each region is indicated in each bar. Note the change in scale of the Y-axis between the soma versus other compartments. Within the arbor, the largest mitochondria are localized within 50 µm of the cell center (* *p* ≤ 0.026), relative to the two other arbor regions, which are not significantly different from each other. (**D**) Bar graph of mitochondrial volume as a function of arbor classification. Mitochondrial sizes are not significantly different in a branch (Br) and the point at which that branch splits into new branches (Branch Point-BrP), but there is typically a significant decrease in size between the proximal branch point and the distal branches (** *p* = 0.0002, * *p* = 0.016, n.s. = not significant).

**Figure 5:**
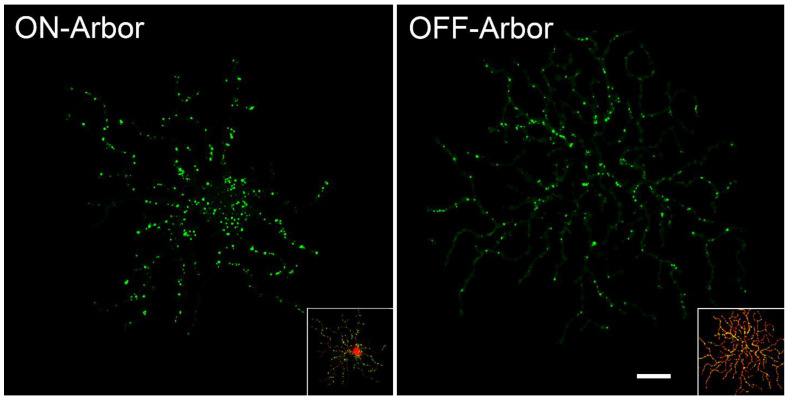
Mitochondrial density in the arbors of a single ON-OFF RGC. Masked out ON and OFF arbors of a single cell showing the distribution of CFP-labeled mitochondria in each (Imaris rendering). The inset shows each arbor (and cell soma with the ON arbor) with the tdTomato cell fill label included. The OFF arbor is larger and more complex, but appears to have a lower density of mitochondria compared to the ON arbor. Scale Bar = 50 µm.

In order to better understand the contributions of dendritic mitochondria in RGC pathology, we have initiated studies to map these organelles in normal conditions and after optic nerve damage. In pilot experiments, we biolistically-labeled individual RGCs in the retinas of Tg-(*Thy1-mitoCFP/COX8*) transgenic mice with a plasmid encoding tdTomato (see Supplemental Methods). The Thy1 promoter drives expression of CFP which is targeted to mitochondria in a subset of RGCs. Figure 4 shows a dual labeled ON-αRGC imaged using a Leica spectral confocal microscope. The 3D images were then imported into Imaris 9.2.1 and used to measure mitochondrial volumes at different regions of the dendritic arbor, classified as “distance” from the soma center and secondarily by classification of different “regions” of the arbor such as primary branch, primary branch point, etc. The soma of this cell contained a wide variety of different sized mitochondria, some that were an order of magnitude larger than in any other region of the cell, while the axon exhibited the smallest mitochondria. Mitochondria could be detected throughout the arbor, even to the distal tips of quaternary branches and there was a correlation between decreasing mitochondrial volume and “distance” with the largest mitochondria being localized within 50 µm from the center of the cell. After branching from the primary arbors, mitochondrial volume decreased significantly to a size that was relatively uniform throughout the rest of the arbor. Importantly, evaluating mitochondrial volume by “classification” showed that mitochondria were typically larger in the proximal region of branch points regardless of whether these were primary, secondary, or tertiary branches. Similar to the ONH, the implication of this pattern of mitochondrial localization is that there is a higher energy requirement to fuel transport at these ramifications. Imaging of an ON-OFF RGC, appears to show that mitochondrial density is higher in the ON arbor relative to the OFF arbor (Figure 5). While further analysis is needed to confirm these observations, it is intriguing to speculate that the increased sensitivity of OFF arbors is correlated to mitochondrial density.

**Figure 6:**
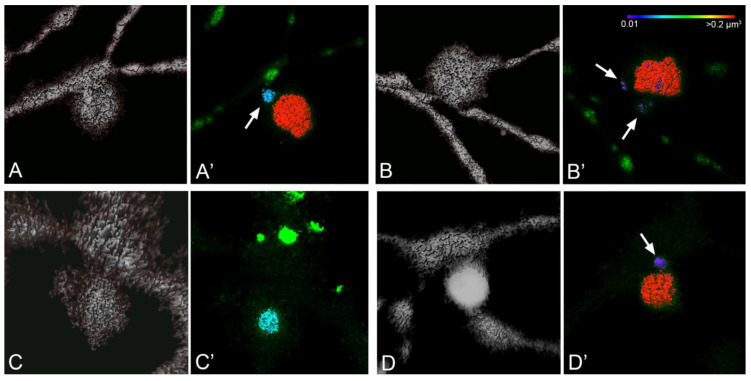
Mitochondrial filled evulsions in dendritic arbors of mouse RGCs. Images of four different evulsions observed in two different RGCs are represented. Images in (**A**–**D**) are Imaris generated surface renderings of each evulsion using the tdTomato cell fill Z-stack, while (**A’**–**D’**) are heat map renderings of mitochondrial volume using the mitoCFP Z-stack in each evulsion, respectively. Each evulsion appears to contain one large mitochondrion and one or more smaller mitochondria (arrows) which are regionally located near the arbor branch where the evulsion has originated. The evulsions are between 5 and 7 µm in axial length. A heat scale relevant for all images is shown in panel (**B’**).

Our pilot studies of mitochondria in the dendritic arbors of RGCs also revealed the presence of putative evulsions that were filled with at least one large and several small mitochondria (Figure 6). These evulsions were larger than the expected size for a dendritic spine, which is a small outpouching of an arbor branch that is a focal site for synapses and also contains at least one adjacent mitochondrion [154]. The nature and the function of these structures requires further investigation, but they may be a second site of transmitophagy in RGCs, although it is likely that they are being phagocytosed by resident microglia as astrocytes do not populate this region of the retina (inner plexiform layer). Consistent with the idea that this is another site of transmitophagy, immunofluorescent staining of healthy retinas revealed SNCG aggregates throughout the inner plexiform layer and an increase in protease resistant SNCG puncta in this region of the glaucomatous retina of DBA/2J mice [144].

## 6. The Role of Mitochondria in Optic Nerve and RGC Pathology

There is little dispute in the literature that mitochondrial function and dynamics play an important role in RGC health and development of the optic nerve [157]. This is fundamentally evident in studies that have linked mutations in genes involved in mitochondrial dynamics and mitophagy with spontaneous optic neuropathies and susceptibility to normal tension glaucoma. As discussed earlier in this review, OPA1 plays an essential role in modulating mitochondrial fusion by regulating and stabilizing cristae formation, and mutations in human OPA1 are associated with dominant optic neuropathy. The pathology to RGCs induced by loss of OPA1 function has been modeled in *Opa1*^+/−^ heterozygous mice, which exhibit a 50% reduction in OPA1 protein levels in the retina. While these mice do not exhibit a loss of RGCs as they age, the dendritic arbors of ON-center RGCs begin to retract, decreasing both their complexity and density of the post-synaptic marker PSD95 [158,159]. Additionally, aged *Opa1*^+/−^ mice show signs of optic nerve axon degeneration and reduced retrograde axonal transport, concomitant with an impaired optimotor response and reduced visual evoked potentials [160,161].

**Figure 7:**
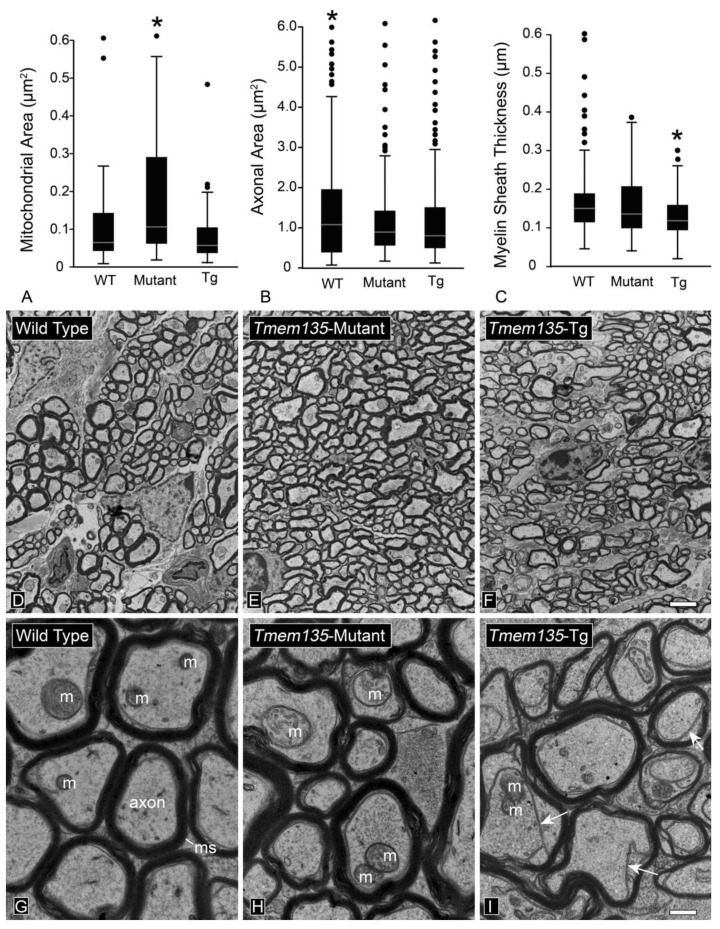
Examination of the optic nerves and RGC mitochondria in mice after modulation of *Tmem135* expression. TMEM135 plays a role in mediating mitochondrial fission [162]. To examine the effect of modulating expression of *Tmem135*, we compared the optic nerves of wild type (WT), *Tmem135* mutant, and *Tmem135* over-expressing (*Tmem135-Tg*) mice at seven months of age using transmission electron microscopy (see supplemental methods). (**A**) Box-and-whisker plots (plotted outliers represent values outside the ninetieth percentile) of mitochondrial area inside axons. Mutant mice exhibited significantly larger mitochondria in RGCs, consistent with a defect in mitochondrial fission in these animals (* *p* < 0.0001, relative to both other genotypes). Transgenic mice exhibited slightly smaller mitochondria than WT mice, but this was not significant. (**B**) Axon areas are larger in WT mice compared to both other groups (* *p* < 0.009). (**C**) Transgenic mice exhibited thinner myelin sheaths than the other two groups of mice (* *p* < 0.0001). (**D**–**F**) Low power representative images of axons in WT, mutant, and transgenic mice, respectively. Scale bar = 2 µm. (**G**–**I**) High magnification images of axons in each group. Mitochondria (m) and myelin sheaths (ms) are indicated. Arrows in (**I**) indicate the membranes of axons that have separated from the myelin sheath in the *Tmem135* overexpressing mice. Scale bar = 500 nm.

The sensitivity of optic nerve architecture in response to the modulation of mitochondrial dynamics can also be illustrated in recent studies done by our group evaluating the optic nerves of FUN025 mice, which carry a mutation in the mouse *Tmem135* gene. *Tmem135* is a putative five trans-membrane domain protein that was identified in an N-ethyl-N-nitrosourea (ENU) screen for age-related defects in mouse retinas [162]. Localization studies showed that TMEM 135 protein co-localized as puncta with mitochondria [162]. Comparison of mouse primary fibroblasts from wild type (WT), the *Tmem135* mutant, and a transgenic mouse over-expressing WT *Tmem135* revealed over-fused mitochondrial networks in *Tmem135* mutant cells whereas cells over-expressing *Tmem135* exhibited over-fragmented mitochondrial networks indicating that this protein played a role in regulating mitochondrial fission, although the exact molecular mechanism is still unknown [162]. Similar observations were also made in vivo. Enlarged mitochondria were observed in the RPE and photoreceptor cells of *Tmem135* mutants [162], while significantly smaller mitochondria were found in the RPE [163] as well as the hearts [164] of mice over-expressing *Tmem135*. Mitochondrial energy production may be also affected in these mice since mitochondrial oxidative phosphorylation proteins were increased in the retinas and hearts of *Tmem135* mutants while their decrease was observed in the same tissues of the over-expressors [163]. Evaluation of mitochondrial sizes in the axons of the myelinated optic nerve showed that mutant mice exhibited impaired fission leading to significantly larger mitochondria, while over-expressing transgenic animals trended toward smaller mitochondria compared to WT littermates (Figure 7). Axon structure was also influenced by TMEM 135. Both mutant mice and over-expressors exhibited significantly smaller axons, while the axons in the over-expressors had significantly thinner myelin sheaths. More surprisingly, however, mice over-expressing TMEM 135 exhibited separation of the myelin sheath from some axons (Figure 7), an architecture that has also been reported in models of Charcot–Marie–Tooth syndrome [165] and in the optic nerves of *Plp^null/y^* mice, a model of spastic paraplegia [166]. In the latter, myelination defects were found to be associated with lower axonal levels of ATP [166], consistent with the evidence that *Tmem135* over-expressors may have reduced capacity for oxidative phosphorylation.

**Figure 8:**
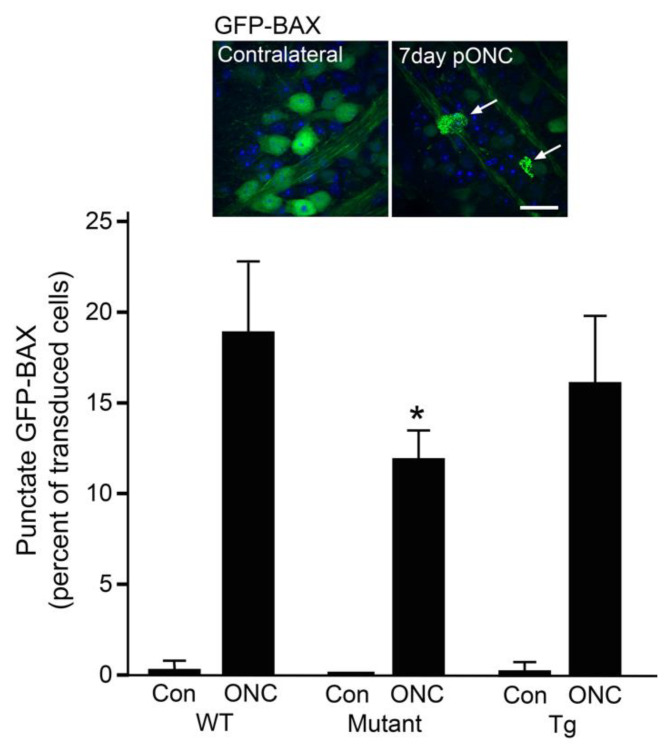
RGCs with larger mitochondria exhibit a reduced level of BAX recruitment to mitochondria after optic nerve damage. RGCs undergo intrinsic apoptosis in response to optic nerve damage, which is dependent on the pro-apoptotic function of the protein BAX. During this process, BAX translocates from the cytosol to the mitochondrial outer membrane and forms bright puncta that are associated with the formation of large molecular weight BAX oligomers [68]. The recruitment of BAX to the mitochondria during apoptosis in RGCs can be monitored by preloading them with a GFP-BAX fusion protein using AAV2/2 viral mediated gene transfer. To test if the modulation of mitochondrial dynamics exhibited by *Tmem135* mutant and *Tmem135* overexpressing transgenic mice (Tg) affected BAX recruitment, we preloaded the retinas of these mice with the GFP-BAX fusion protein and then subjected them to optic nerve crush surgery (see Supplemental Methods). At seven days post-crush surgery, the retinas from both crush and contralateral eyes were scored for punctate BAX localization as a percentage of transduced cells. The inset shows transduced RGCs from the contralateral retina and crush retina of a wild type (WT) mouse, with arrows highlighting cells with punctate GFP-BAX. Scale bar = 25 µm. The bar graph shows that *Tmem135* mutant mice still exhibit cells with punctate GFP-BAX in crush retinas, but the level is lower than in the other genotypes (* *p* = 0.013).

At this stage, it is not clear that abnormally high steady-state levels of mitochondrial fission confer increased susceptibility to RGCs under stress conditions. We did not find an increase in the rate of GFP-BAX recruitment to mitochondria after optic nerve crush surgery in over-expressing mice, compared to WT littermates. We did find, however, a significant delay in BAX recruitment in *Tmem135* mutant animals, suggesting that a condition with larger and more networked mitochondria is partially protective (Figure 8). Consistent with these observations in *Tmem135* mutant mice, mice treated with the DRP1 inhibitor Mdivi-1 exhibited larger mitochondria and increased protection of RGCs in ischemia-reperfusion injury [167], while AAV2-mediated gene transfer of the dominant-negative DRP1^K38A^ mutant reduced RGC soma loss and axon degeneration in early stage glaucoma (nine months of age) in DBA/2J mice [168].

Mouse models have also been used to evaluate the effects of OPTN and TBK1 in the retina and optic nerve. *Optn*^−/−^ mice are generally normal with the exception that they have a reduced ability to clear bacterial infections [169]. Transgenic mice expressing the E50K mutation of *OPTN*, however, demonstrate an optic neuropathy that is associated with an increase in mitochondrial fission and mitophagy [170,171], along with visual impairment [172]. While mutations in human OPTN have also been linked to ALS, the mechanism of the pathology appears to act independently between the two diseases. Mutations that cause optic atrophy and are associated with glaucoma are not associated with ALS and vice versa. ALS-causing mutations in OPTN inhibit Parkin-dependent mitophagy, while glaucoma-causing mutations do not [173]. Conversely, glaucoma-causing mutations stimulate the death of 661W retinal precursor cells but not in a motor neuron cell line, while ALS-causing mutants had no effect in 661W cells but were cytotoxic to the motor neuron cells [174]. Surprisingly, the E50K mutant was able to rescue mitophagy in OPTN deficient HeLa cells overexpressing Parkin [173], leading to speculation that OPTN mutations that impart RGC and optic nerve pathology may actually be a gain-of-function leading to overstimulation of mitophagy. Alternatively, other studies have suggested E50K-induced pathology is not directly related to mitochondrial dynamics. Instead, Chi and colleagues [170] found that the E50K mutant disrupted normal interactions between WT OPTN and Rab8 GTPase, a protein that is involved in multiple cell functions associated with vesicle trafficking and autophagy [175]. Through this mechanism, the E50K mutant protein was shown to inhibit the autophagic flux of cells leading to death, a process that could be blocked by the autophagy inducer rapamycin [176]. Given the wide variety of cell functions regulated by Rab8 and OPTN, however, there is still some uncertainty of how OPTN glaucoma-causing mutants function to cause RGC death.

Similar to OPTN, TBK1 has been linked to familial forms of normal tension glaucoma, and ALS combined with frontotemporal dementia (FTD) [32,33,177]. Unlike the glaucoma-causing mutation of *TBK1*, which is actually a gene duplication leading to overexpression, ALS-FTD mutations disable TBK1 function, underscoring the complex pathologies that can be induced by altering normal function of the TBK1-OPTN axis. Increasing TBK1 activity also affects autophagy in cells. Fibroblasts and retinal ganglion-like cells derived from induced pluripotent stem cells from a normal tension glaucoma patient with the *TBK1* duplication, exhibited an intrinsically higher autophagic flux compared to control (WT) cells [178], while transgenic mice expressing one or two copies of the human *TBK1* gene demonstrate an age-related loss of their RGCs that is independent of ocular hypertension [179]. To date, the effect of TBK1 overexpression in RGCs and its impact on mitophagy or mitochondrial dynamics has not been investigated.

One of the most striking examples of mitochondrial dysfunction and selective sensitivity of RGCs is characterized by Leber’s hereditary optic neuropathy (LHON). LHON was originally reported in 1871 by Theodor Leber as painless visual loss in young adults from several families that exhibited a pattern of maternal transfer and a bias toward affecting males. Over 100 years later, the first genetic mutation associated with LHON was described in the mitochondrial encoded gene for NADH Dehydrogenase 4 (*MTND4*) and subsequent mutations causing LHON have been reported in the *MTND1* and *MTND6* genes as well. Collectively, between 90 and 95% of all LHON cases are linked to mutations in these genes (reviewed by [180,181,182]). The *MTND* genes encode subunits that are part of respiratory Complex I in the electron transport chain. Similarly, families with autosomal recessive LHON (arLHON), which is not maternally transferred, have been found to carry mutations in the nuclear-encoded gene, *DNAJC30*. DNAJC30 has been implicated as a chaperone protein that shuttles damaged subunits of Complex I away for turnover [183]. With these links to Complex I, it is not surprising that the underlying pathology of LHON has been suggested to be tied to inadequate ATP generation and increased ROS production, both due to inefficient electron/proton transport across the MIM. What is surprising, however, is that while all mitochondria in an affected individual carry one of these mtDNA mutations, only the RGCs and optic nerve are primarily affected. Additionally, LHON, including arLHON, exhibits variable penetrance, with approximately 50% of male and 10% of female carriers losing vision, usually as young adults. The implication from the variable penetrance is that other factors, including environmental ones, play a role in pushing vulnerable RGCs toward degeneration [181,182]. This may be true, but if so, it is astounding that mtDNA and nuclear genes associated with LHON are not frequently identified as risk factors for glaucoma.

While many of the genetic associations between mitochondria and optic nerve pathology have been derived from studies of familial forms of glaucoma (and optic atrophy), there has only been a single locus identified as a risk allele in genome-wide association studies involving patients with more genetically complex, and more prevalent, forms of primary open-angle and normal tension glaucoma. This locus, which contains the gene for mitochondrial thioredoxin reductase (*TXNRD2*), was detected in a meta-analysis of eight independent genome-wide association studies from the United States, Australia, Europe, and Singapore [184]. Rare mutations in *TXNRD2* have been detected in other complex human diseases, principally cardiomyopthies [185]. TXNRD2 functions to modulate cellular redox balance and ROS scavenging, which likely is important for homeostasis in cells that rely heavily on mitochondrial oxidative phosphorylation such as neurons and cardiac myocytes. Immunolocalization studies in the mouse eye suggest that TXNRD2 protein expression was enriched in RGCs and astrocytes of the ONH [184]. A further analysis of mitochondrial involvement in complex forms of glaucoma was also conducted by inter-sectional data-mining of the MitoCarta database, which is a catalog of all known protein coding genes that are localized to mitochondria (broadinstitute.org), the Kyoto Encyclopedia of Genes and Genomes (KEGG), and glaucoma-associated genes, to identify KEGG pathways that reflect mitochondrial genetic variation [186]. Pathways that specifically support a role for mitochondria in the pathogenesis of glaucoma were identified and include several associated with lipid and carbohydrate metabolism.

Significant changes in mitochondrial dynamics and function occur in the ONH of DBA/2J mice during the progression of glaucoma. In the MTZ, mitochondria become more fragmented and appear to lose cristae structure [168,187], which is associated with the release of OPA1. Recent studies suggest that glaucomatous RGCs lose function of A-kinase anchoring protein 1 (AKAP-1) [188], which normally facilitates the phosphorylation of Ser637 on DRP1 leading to its inactivation [189]. Mice lacking *Akap1* exhibit fragmented mitochondria [188] and at least some of the mitochondrial defects in the ONH of DBA/2J mice can be rescued by gene transfer of the K38A dominant negative mutant of DRP1 [188]. At this point, it is not clear if the structural changes in mitochondria observed in the ONH of DBA/2J mice are promoting glaucomatous damage in this model, or are a consequence of intrinsic apoptosis. Indeed, these changes are reported to occur in concert with BAX-mediated changes, suggesting the latter.

Other studies have investigated changes in mtDNA in peripheral lymphocytes of patients with primary open-angle, angle-closure, and exfoliation glaucoma. These studies reported polymorphisms, some considered “potentially pathogenic”, in these patients that were not found in age-matched control subjects [190,191,192]. None of the polymorphisms were detected in the *MTND* genes associated with LHON. The same group found that the respiratory function of peripheral lymphocytes was also decreased [190], suggesting that reduced mitochondrial function was systemically intrinsic in individuals who develop the disease. Important studies have also evaluated the respiratory capacity of ONH tissue by SeaHorse analysis, from aging DBA/2J mice in which the contributions of astrocytes were suppressed using the glial-specific aconitase inhibitor fluorocitrate [193]. Aging ONHs from both a genetically controlled (non-glaucomatous) strain (DBA/2J-*Gpnmb*^+^) and DBA/2J mice exhibited decreases in maximal respiration, ATP production, and spare capacity, with lower maximal respiration exhibited in the glaucomatous mice. Interestingly, O_2_ consumption and ATP production was increased in glaucoma, but not as a product of increased glycolysis. These important studies provide some insight into the energy requirements and the metabolic response of RGC axonal mitochondria during glaucomatous pathology.

## 7. A Bioenergetic Model of Glaucomatous Pathology

The cumulative knowledge of mitochondrial function, distribution, and pathology is shaping a narrative of the metabolic contributions to the pathogenesis of RGC susceptibility and glaucoma. It is clear, from the study of familial diseases, that RGCs are highly sensitized to mutations that affect mitochondrial dynamics and respiratory function. The distribution of RGC mitochondria, with the highest density being localized to the axons of the ONH, suggest that this region is highly energy dependent and, therefore, exquisitely sensitive to alterations in metabolic capacity and support. While we suspect that ocular hypertension-mediated strain increases at the ONH and leads to an increase in metabolic demand, we currently do not have a complete understanding of how this demand is compensated for. One hypothesis is that hypertension-mediated changes in ONH remodeling [121,122,124,194] shift the attention of supporting cells away from their normal metabolic support of the axons. During the progression of glaucoma, studies in mice showed that astrocytes change behavior and morphology [125,126,127] and, at least early in the disease, provide an important protective function for axons [195]. More recent studies indicate that astrocytes change their orientation to axonal fibers and form a syncytium interconnected by gap junctions [196], which allows them to redistribute glycogen stores to areas of high demand [197]. Taken together, an elegant model of adaptive responses based on energy demand and resource redistribution was recently outlined [198]. This model eventually predicts that the adaptive responses of the astrocytes provide only a transient protective effect, and eventually are unable to sustain the energy demand over a long period of time. Ultimately, RGC axons are expected to reach an energy crisis that is likely manifested as a decrease in axonal transport mechanisms, leading to further pathology both proximal and distal to the ONH. It seems reasonable to assume that the threshold at which this energy crisis is reached is intimately linked with how robust the RGC mitochondria are when the challenge is presented.

## 8. Therapies Targeting Mitochondria in RGCs

A variety of therapies are being developed that are focused on preserving or improving mitochondrial structure and function. With respect to RGCs and the optic nerve, small molecule supplements of antioxidants and electron donors or acceptors have shown promise as neuroprotective agents for glaucoma or acute and ischemic optic nerve damage. The electron carrier coenzyme Q10 (coQ_10_), with or without vitamin E, has been demonstrated to improve mitochondrial function and preserve RGC numbers in several different models of optic nerve disease, including experimental glaucoma [199,200,201]. The success of these experiments has prompted the development of a clinical trial testing the efficacy of CoQun, an eye drop mixture of CoQ_10_ and vitamin E in patients with primary open-angle glaucoma [202]. Similarly, nicotinamide (niacin, vitamin B3) supplementation has been shown to dramatically attenuate axonal degeneration in the DBA/2J mouse model of glaucoma [203,204]. The mechanism of action of these small molecules, directly on mitochondrial function, is not entirely clear. CoQ10, while being a critical electron transporter in oxidative phosphorylation, is also a powerful antioxidant localized to the MIM and may provide protection against lipid peroxydation of this membrane as a normal function of aging [205]. Nicotinamide is the precursor of the redox reaction cofactors NAD^+^ and NADP^+^ and, therefore, may be indirectly protective as an antioxidant. However, axon degeneration is associated with NAD^+^ depletion as a function of the enzyme sterile alpha and TIR motif containing one (SARM1) protein [206]. Some byproducts of SARM1 activity (ADP-ribose and cyclic ADP-ribose) are associated with Ca^2+^ release from the ER and mitochondria [207] leading to the subsequent activation of calcium-sensitive proteases such as calpains (reviewed by [208,209]). Rather than directly affecting mitochondria, nicotinamide promotes increased levels of NAD^+^, which in turn inhibit SARM1 activity [210].

There is also growing interest in the efficacy of small mitochondrial-targeted peptides such as elamipretide (SS-31). Elamipretide has a high affinity for cardiolipins and becomes concentrated in the MIM, helping to stabilize cristae structure and improve mitochondrial function and transport, properties that have been touted as being able to reverse the effects of aging on mitochondria [211,212]. Studies also show that it accelerates the formation of reticulated mitochondria in tissue culture cells [213]. Based on these effects, elamipretide has been tested as a small molecule in several neurodegenerative models, showing promise. Systemic delivery of elamipretide reduced RGC loss and improved cell function in both a rat model of experimental glaucoma [214] and acute optic nerve trauma [215]. Although elamipretide has an affinity for cardiolipins, suggesting that it may antagonize the action of DRP1-mediated cardiolipin MIM to MOM transfer during apoptosis, several lines of evidence suggest that elamipretide does not phenocopy DRP1 deletion or inhbition [213]. In fact, combined therapy using the DRP1 inhibitor, Mdivi-1, and elamipretide has been shown to be synergistic [216].

A growing area of interest for enhancing cell resilience in disease is mitochondrial transplantation. Here, healthy mitochondria derived from a donor source are introduced directly into cells with reduced mitochondrial function (reviewed by [217,218]). Studies have shown efficacy of both autologous and non-autologous transfers in a variety of neurodegenerative conditions even with simple intravenous delivery of isolated mitochondria [219]. Mitochondrial transplantation into RGCs has recently been reported [220]. In these experiments, mitochondria were purified from rat livers, labeled with mitoTracker red, and injected intravitreally into rat eyes. Within a day, investigators were able to visualize labeled organelles in tubulin positive RGCs in the retina and show improved respiratory capacity and electrophysiological responses. Importantly, the mitotherapy provided a modest increase in RGC survival and regenerative capacity in a model of acute optic nerve damage. The concept of mitotherapy to bolster RGCs is attractive given the high accessibility of these cells by intravitreal injection. Many questions remain unanswered, however, including knowledge of whether or not transplanted mitochondria are able to persist and replicate as well as a need to improve the field size of transplantation to increase the numbers of cells receiving the donor mitochondria.

In summary, therapeutic efforts to scavenge free radicals, increase respiratory capacity, or enhance mitochondrial fusion (by modulating the activity of DRP1) have shown promise in increasing the resiliency of RGCs to optic nerve damage.

## Data Availability

Raw data is available from the authors upon reasonable request.

## Supplementary Materials

The following is available online at https://www.mdpi.com/article/10.3390/cells10071593/s1, Supplementary Methods.
