# Supplementary material for: The Influence of Mitochondrial Dynamics and Function on Retinal Ganglion Cell Susceptibility in Optic Nerve Disease
Source: Cells. 2021 Jun 25;10(7):1593. doi: 10.3390/cells10071593 (PMC8306483; doi:10.3390/cells10071593)
Supplement: Supplementary file 1 [file cells-10-01593-s001.zip › cells-1247482-SI.pdf]

Supplemental Methods

Mouse Optic Nerve Head Immunostaining

Mice were euthanized by an intraperitoneal injection of a lethal dose of euthasol. Eyes were enucleated and fixed in 4% paraformaldehyde in PBS (50 mM phosphate buffer with 150 mM NaCl, pH 7.4) at room temperature for 50 minutes and then transferred into 0.4% paraformaldehyde in PBS and stored over night at 4˚C. The anterior chamber and lens was then removed and the eyes equilibrated in 30% sucrose in PBS at 4˚C overnight. After washing in PBS, the eye cups were oriented in Tissue-Tek optimal cutting temperature (OCT) compound (ThermoFisher Scientific, Waltham, MA) and frozen on dry ice. Sections (10 µm thick) were cut and ones containing the optic nerve head in profile were collected. For staining, sections were thawed and then blocked in PBS containing 2% BSA and 0.1% Triton X-100 overnight at 4˚C. Blocked sections were overlaid with block buffer containing a mouse monoclonal antibody against TOM20 (clone 4F3, 10 µg/mL dilution, Sigma-Aldrich, St. Louis, MO) overnight at 4˚C in humidified chambers and then washed in PBS followed by another overnight incubation with block buffer containing a goat anti-mouse IgG conjugated to Texas Red (1:1000 dilution, Jackson Immunoresearch, West Grove, PA). After further washing, the sections were cover slipped with a drop of Vectashield containing DAPI to stain nuclei (Vector Laboratories, Burlingame, CA). Color Digital images were photographed with a Zeiss AxioImager.A2 epifluorescent microscope and a 10X objective lens (Carl Zeiss MicroImaging, Inc., White Plains, NY).

Mitochondrial Analysis in the Dendritic Arbors of Retinal Ganglion Cells

Transgenic mice expressing Thy1-mitoCFP (B6.Cg-Tg(Thy1-CFP/COX8A)$^{S2Lich/J}$, The Jackson Laboratory, Bar Harbor, ME), which allows for expression of CFP in retinal ganglion cell mitochondria, were euthanized and retinas were extracted. The retinas were then whole mounted on filter paper and placed in cold oxygenated artificial cerebrospinal fluid (ACSF - 119 mM NaCl , 2.5 mM KCl, 2.5 mM $CaCl_2$, 1.3 mM $MgCl_2$, 1 mM NaH_2PO_4, 11 mM glucose, and 20 mM HEPES, pH 7.4). A Helios Gene Gun system (Bio-Rad Laborratories, Richmond, CA) was used to biolistically label individual retinal ganglion cells using 1.6 µm gold particles coated with a CMV-tdTomato expressing plasmid. Retinas containing the tdTomato labeled retinal ganglion cells were incubated in cold oxygenated ACSF for 24 hours. After incubation, the retinas were fixed at room temperature for 20 minutes in 4% paraformaldehyde in PBS and then washed in PBS. Retinas were then mounted onto slides, cover slipped, and imaged with a Leica TCS SP8 confocal microscope (Leica Microsystems Inc., Buffalo Grove, IL). The resulting images were 3D stacks of between 126 and 154 optical sections, containing an entire ganglion cell arbor complex with both the tdTomato (cell fill) and mitoCFP (mitochondria) channels.

Following imaging, the stack file for each labeled cell was separated into its respective channels (tdTomato cell fill and Mito-CFP) using ImageJ. The cell fill file was uploaded to Amira 2019.4 (ThermoFisher, Waltham MA) and masked using Amira's 'magic wand' tool to ensure that only pixels corresponding to the cell of interest were included in downstream analyses. The mitoCFP file of the same cell was then uploaded to the Amira project file, and mitochondrial signal specific to the masked cell of interest was extracted using the "A*B" arithmetic function, which uses the masked cell pixels/area from the cell fill to determine which mitochondrial signal is extracted. The stack containing the extracted mitoCFP signal and the stack containing the masked out cell fill were then merged on ImageJ to create a 3D composite image with both the mitoCFP and tdTomato cell fill signals. For analysis of the arbors of a single ON-OFF retinal ganglion cell, each arbor was masked as a separate file.

The new composite file was converted to a .ims (Imaris file) format using the Imaris File Converter x64 9.2.1 (Oxford Instruments, Abingdon UK) and uploaded to the Imaris arena. Utilizing the Imaris 'measurements' tool, measurement points from 50 µm , 100 µm , and 150 µm from the center of the cell soma were marked in concentric circles around the cell. Regions of interest relating to the 50, 100, and

150 µm measurements were created using the Imaris 'surfaces' function and mitochondrial volume was analyzed with a standard set of parameters. Heat map images were also generated using the 'surfaces' function, following mitochondrial volume analyses. Data from this analysis was exported to GraphPad Prism (9.1.1) software for statistical analyses.

Analysis of *Tmem135* mice

Wild type (WT), *Tmem135* mutants, or *Tmem135* overexpressing transgenic mice (C57BL/6 genetic background, n=4 mice each genotype) at 7 months of age were intravitreally injected with 1 µL of AAV2/2-*Pgk*-GFP-BAX virus (at a minimum of 10^{12} genome copies/mL, packaged by University of North Carolina Vector CORE, Chapel Hill, NC) as previously described [1]. Four weeks later, optic nerve crush was performed to induce RGC apoptosis [2]. After 7 days the animals were euthanized and the eyes, with approximately 2 mm of adjoining optic nerves, were enucleated. Optic nerves were removed and placed in phosphate buffer (PB; 50 mM, pH 7.4) containing 2.5% glutaraldehyde and 2% paraformaldehyde and stored at 4˚C until processed for electron microscopy. The globes were fixed as described above. Retinas were then isolated, flat mounted with the ganglion cell layer facing up and the retina divided into 4 equal quadrants, and counter stained with DAPI (300 ng/mL) for 10 minutes before being cover-slipped. Digital images of the peripheral to mid-peripheral regions of all 4 quadrants were collected using a Zeiss Axioimager Z2 upright microscope (Carl Zeiss MicroImaging, Inc.) using a 20X objective lens. BAX recruitment was assessed by quantifying the percentage of transduced RGCs exhibiting punctate GFP-BAX.

The optic nerves from the uncrushed eye (n=4 per genotype) were processed further by first being post-fixed in 1% osmium tetroxide in PB, then dehydrated through an ethanol series and embedded in Epon epoxy. Sections (60-90 nm) were cut through the myelinated region of the nerve, stained with 50% ethanoic uranyl acetate and Reynold's lead citrate. Sections were viewed using a Phillips CM120 transmission electron microscope (FEI Company, Hillsboro, OR). For metric analysis, digital images were imported into ImageJ (v1.42q) and the areas of both the axons and mitochondria within axons were measured. The average thickness of the myelin sheaths of axons present were also measured. Overall, data was collected from 943, 1098, and 1141 axons from WT, *Tmem135* mutant, and *Tmem135* transgenic mice, respectively.

References

1. Donahue RJ, Maes ME, Grosser JA, Nickells RW: BAX depleted retinal ganglion cells survive and become quiescent following optic nerve damage. Mol Neurobiol 2020, 57:1070-1084.
2. Li Y, Schlamp CL, Nickells RW: Experimental induction of retinal ganglion cell death in adult mice. Invest Ophthalmol Vis Sci 1999, 40:1004-1008.
